# Optimization of Lithium‐Mediated Nitrogen Reduction via Electrolyte Engineering

**DOI:** 10.1002/EXP.20240515

**Published:** 2026-07-15

**Authors:** Xiyang Cai, Kaixiang Wang, Ruilin Wang, Liuxuan Luo, Shuiyun Shen, Guanghua Wei, Zhichuan J. Xu, Junliang Zhang

**Affiliations:** ^1^ Institute of Fuel Cells, School of Mechanical Engineering Shanghai Jiao Tong University Shanghai China; ^2^ School of Materials Science and Engineering Nanyang Technological University Singapore Singapore; ^3^ National Engineering Research Center of Chemical Fertilizer Catalyst (NERC‐CFC), School of Chemical Engineering Fuzhou University Fuzhou China; ^4^ Paris Elite Institute of Technology Shanghai Jiao Tong University Shanghai China

**Keywords:** ammonia synthesis, lithium‐mediated nitrogen reduction, electrolyte engineering, composition regulation, non‐liquid electrolytes

## Abstract

Ammonia serves as a fundamental precursor for nitrogen‐based chemicals in both agriculture and industry, which is mainly produced by the Haber–Bosch process. Lithium‐mediated nitrogen reduction (LiNR) has emerged as a promising alternative to the Haber–Bosch process, though further optimization of its performance is required. Based on the reaction mechanism of the LiNR process, we contend that electrolyte engineering is a pivotal method for optimization in LiNR and other metal‐mediated nitrogen reduction processes (MNR). By merging insights from both LiNR and battery research, several strategies have been proposed, including composition regulation, development of non‐liquid electrolytes, and decoupling the catholyte and anolyte. The perspective highlights the critical role of electrolyte engineering and inspires further refinement of MNR, contributing to greener and more efficient ammonia synthesis.

## Graphical abstract

Ammonia production today relies on the energy‐demanding Haber–Bosch process. By optimizing lithium‐mediated nitrogen reduction through electrolyte engineering, this work outlines strategies to make ammonia synthesis cleaner, more efficient, and more sustainable.

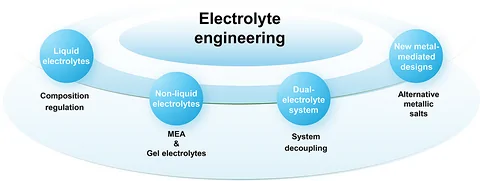

## 1 Introduction

Ammonia is a raw material for a multitude of nitrogen‐containing chemicals utilized in both agricultural and chemical industrial processes. The current global production of ammonia reaches 180 million metric tons per year [1], with projections suggesting a continued growth trajectory in the future. The expected increase in production is attributed to its potential serving as carbon‐free energy carrier with high mass fraction of hydrogen [2]. The Haber–Bosch process, served as the principal method of ammonia production, is characterized by the high temperature (350–450°C) and the high pressure (150–200 atm), as well as the massive carbon dioxide emissions associated with fossil‐based steam reforming process [3]. Electrochemical ammonia synthesis has been extensively investigated due to its mild reaction conditions and the potential for decentralized production. It also facilitates green ammonia synthesis by the integration of renewable energy sources, thus recognized as an alternative to the thermochemical process.

In the field of nitrogen fixation at ambient temperature and pressure, lithium‐mediated nitrogen reduction (LiNR) has emerged as the only reproducible electrochemical system for ammonia synthesis from nitrogen reduction at ambient conditions. After instituting stringent verification methodologies [4], numerous research groups have successfully replicated the experiments, thereby affirming the system's reliability. Differentiated from thermal catalysis process, photocatalytic process, or other electrocatalytic process that rely on dissociative or associative pathway of nitrogen fixation [5, 6, 7], LiNR utilizes metallic lithium to mediate the nitrogen dissociation process. The strategy is effective because the reaction between metallic and nitrogen is thermodynamically favorable in ambient conditions. Meanwhile, the electrolytic system based on an organic solvent was demonstrated to substantially retard the competing hydrogen evolution reaction (HOR). The concept was initially proposed by Fitcher et al. in 1930 [8], and a prototype for subsequent studies was designed in 1990s by Tsuneto et al. [9, 10]. In recent years, other metal‐mediated reaction systems (including calcium‐mediated [11] and magnesium‐mediated [12]) have been proposed, but the reliability requires further independent verification from other groups.

**FIGURE 1:**
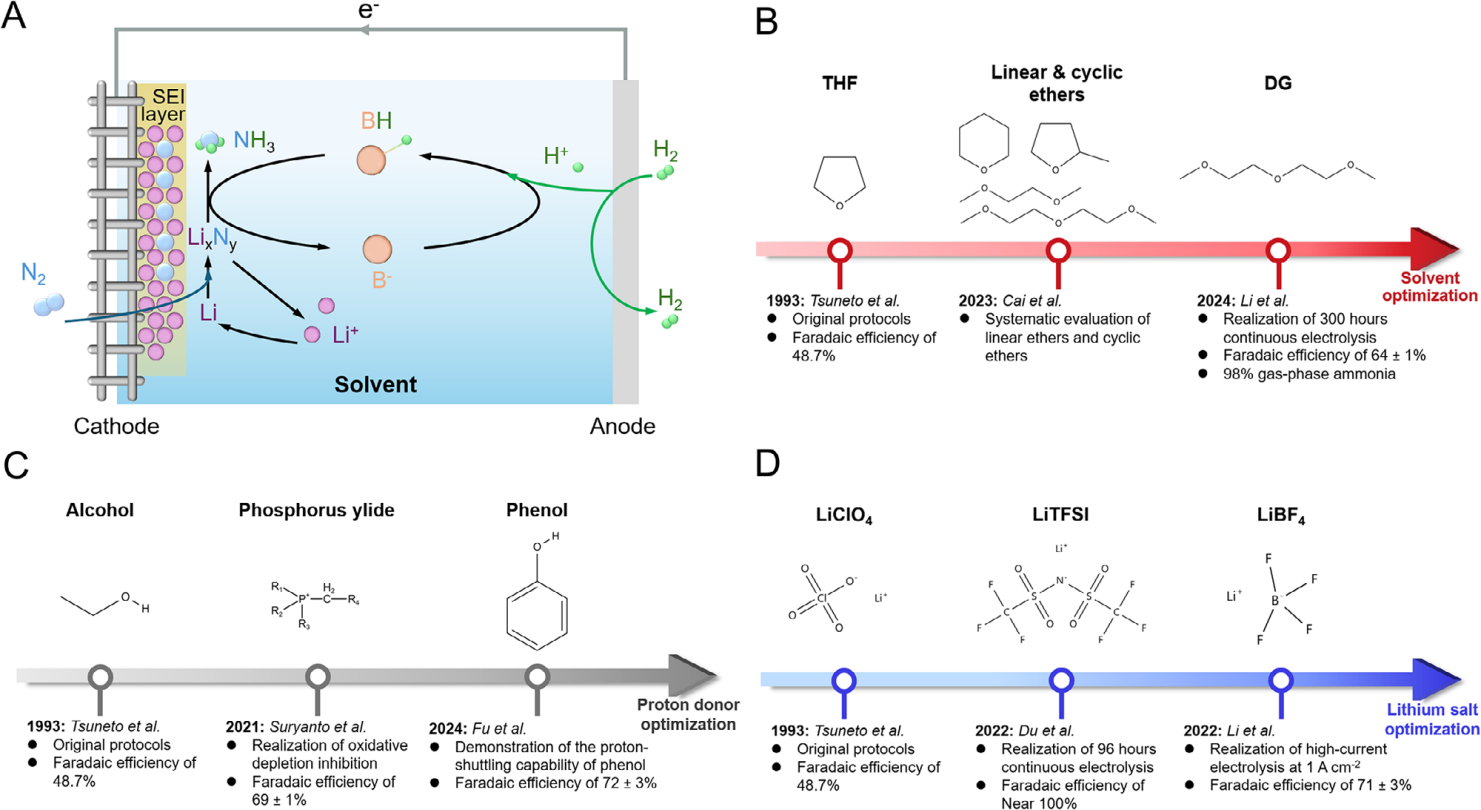
(A) The reaction mechanism of LiNR, BH stands for proton donor. (B–D) The technological routes of (B) solvent, (C) proton donor, and (D) lithium salt in LiNR study, shown by representative works in this field. THF and DG are short for tetrahydrofuran and diglyme, respectively.

Even though the precise mechanism of the reaction hasn't been fully understood, it is generally accepted that the LiNR proceeds through three steps. As illustrated in Figure 1A, the process commences with the electro‐deposition of solvated lithium ions on the electrode surface, followed by the combination of lithium metal with nitrogen to form lithium nitride, on which ammonia is synthesized with protons delivered by the proton donor (BH), accompanied by regeneration of lithium ions.

So far, the uniqueness and effectiveness of LiNR have been widely recognized but it still faces a series of problems in terms of practical applications, including plateaued ammonia yield rate and faradaic efficiency, limited durability, and low energy efficiency. Given that LiNR is an interfacial electrochemical reaction, its performance should be dictated by electrode and electrolyte. Nevertheless, the mechanism diagram verifies that the LiNR process is closely associated with electrolyte components (namely, lithium salt, proton donor, and solvent), highlighting the importance of regulating the electrolytes for LiNR. Hence, a series of studies have investigated the specific effects of the electrolyte components on the reaction, though mainly based on liquid electrolytes. It is reported that solvent affects conductivity and selectivity of the system, which is realized by regulating the solvation state of lithium ions and the composition of SEI layer [13, 14]. Besides, the stability of the system is also suggested to be closely associated with the solvent [14]. On the other hand, the lithium salt and the proton donor participate in the deposition and protonation steps (Figure 1A), which could be the rate determining rate steps of the LiNR process [15, 16]. To construct an efficient LiNR system with high ammonia yield, a fully dissociated and stable lithium salt in combination with moderate proton donor is essential. It should be noted that the compatibility of the electrolyte components should also be considered, as they collaboratively determine the composition and properties of solid‐electrolyte interphase (SEI), a passivation film produced by the reaction between metallic lithium and electrolyte that significantly affects LiNR performance [15, 16].

In this vein, the optimization of the LiNR fundamentally involves the comprehensive regulation of the three primary components in the electrolyte. It is anticipated that coordinated electrolyte engineering is pivotal not only for enhancing the performance of lithium‐mediated systems but also for advancing other metal‐mediated processes in general. The strategies for electrolyte engineering are summarized as follows.

## 2 Component Optimizations Based on Liquid Electrolytes

To date, numerous studies have explored the role of solvents, proton sources, and lithium salts, which collectively contributed to the system performance [13, 14, 17, 18, 19, 20, 21]. The solvent optimization work is illustrated in Figure 1B. Cai et al. evaluated several representative linear and cyclic ethers under a variety of operating conditions [13]. Their findings show that solvent molecules influence LiNR performance by dictating the solvation configurations of conductive ions and inducing the formation of solid electrolyte interphase with different compositions. This discovery is echoed by the work reported by Li et al. [14], in which diethylene glycol dimethyl ether (DG) is utilized as the solvent. After 300 h of continuous electrolysis, a faradaic efficiency of 64 ± 1% and a gas phase ammonia content of 98% were obtained, which significantly demonstrates the stability and the optimal product distribution of the system.

In terms of proton donors (Figure 1C), Suryanto et al. devised an electrolytic system utilizing a phosphine ylid as the proton shuttle, resulting in an ammonia yield of 53 ± 1 nmol s^−1^ cm^−2^ and a faradaic efficiency of 69 ± 1%, with the oxidative depletion of the proton donor being suppressed [17]. Based on their experimental findings with multiple sets of proton donors, Lazouski et al. suggest that proton donors may affect the reaction rate by influencing the penetration of the SEI layer and put forward kinetic modeling for this property [18]. Meanwhile, Fu et al. demonstrate a higher suitability of phenol for flow cell, achieving a faradaic efficiency of 72 ± 3% [19].

The impact of various lithium salts essentially originated from different anions, which plays a pivotal role in the formation of the SEI layer during the dissociation process. Du et al. employed LiTFSI (lithium bis (trifluoromethyl sulfonyl) imide) to attain stable ammonia yields of 150 ± 20 nmol s^−1^ cm^−2^ [20]. The faradaic efficiency was close to 100%. Li et al. achieved an ammonia yield of 2500 ± 100 nmol s^−1^ cm^−2^ and a faradaic efficiency of 71 ± 3% with LiBF_4_ at a current density of 1,000 mA cm^−2^ [21]. These two processes are similar in that both utilize fluorinated lithium salts, providing stable Li^+^ channels and stable SEI interfaces for the reaction through the dissociation of LiF (Figure 1D).

Nevertheless, the existing research is still focused on the development of electrolytes comprising a single component. With the broadening spectrum of available options, it is anticipated that multi‐components electrolyte engineering will engender changes to the system by combining the advantages of individual components. Li et al. employed the empirical law of ring‐chain coupling in lithium batteries as a guide to tailor the electrochemical performance of the electrolyte [22]. They paired the cyclic ether with the chain ether to form a hybrid solvent, which amalgamates the electrochemical advantages of both solvents while achieving a high faradaic efficiency of 54.78 ± 1.60%.

The use of additives represents another approach for optimization, leading to counterintuitive results. Although anhydrous and oxygen‐free conditions are theoretically optimal for the reaction, Li et al. realized faradaic efficiencies of 78.0 ± 1.3% by adding 0.6–0.8 mol% oxygen in nitrogen [23]. Similarly, Spry et al. discovered that a water concentration of 35.9 mm resulted in the optimal faradaic efficiency of in their system [24]. It can be deduced that, though only in small quantities, the presence of an additive may impart a positive effect on the reaction system when the concentration is meticulously regulated. This underscores the significance of additives as a feasible strategy for enhancing performance through electrolyte engineering.

Opportunities for optimization also extend to lithium itself. The deposition potential of lithium (−3.04 V vs. the standard hydrogen electrode) leads to the primary challenge to the energy efficiency of LiNR [11]. The replacement of Li^+^ in solution with other cations that exhibit more positive thermodynamic potentials can effectively reduce the system's energy consumption. Nonetheless, the same principles of electrolyte engineering should be followed, particularly the synergistic selection of anions and solvents in designing other metal‐mediated reactions. It should also be noted that beyond affecting the dissociation energy of ionic bonding interactions between anions and cations, anions can also influence the solvation activity of cations and potentially contribute to side reactions. Fu et al. employed Ca[B(hfip)_4_]_2_ (calcium tetrakis (hexafluoroisopropyloxy) borate) in a CaNR system to enhance the solubility of metal salts, ultimately obtaining a faradaic efficiency of 40 ± 2% [11]. In comparison, Krebsz et al. selected a solvent mixture comprising Mg^2+^‐Li^+^‐BH_4_^−^‐ TFSI^−^ to prevent surface passivation during the MgNR, thereby rendering the reaction feasible and ultimately enabling a faradaic efficiency of 7% [12]. These results indicate that lithium replacement is an attractive approach, showing potential for higher energy.

It is worthwhile to mention that although liquid electrolytes represent the most extensively studied system, their liquid nature also introduces a series of issues, such as limited gas transfer, reliance on organic solvents, and significant voltage loss, necessitating further exploration for other types of electrolytes.

## 3 Development of MEA Based on Polymer Electrolyte Membrane

The solubility of nitrogen is between 10^−5^–10^−3^ in most common solvents [25]. The unfavorable nature of nitrogen gas restricts the nitrogen reduction rate by limiting its diffusion. Although this problem can be greatly alleviated by increasing nitrogen pressure, a more effective and energy‐saving method is to integrate the electrode and electrolyte into a thin film to produce membrane electrodes assembly (MEA), facilitating efficient gas transfer, reduced solvent consumption, and compact configuration. It differs from liquid electrolytes in that the organic solvent is replaced by a polymer electrolyte membrane.

Cai et al. designed a set of MEA configuration for LiNR, realizing an ammonia faradaic efficiency of 9% [26]. The advantages of MEA are clear. Their integrated electrode design and independence from liquid electrolytes provide significant electrochemical performance benefits, such as lower voltage loss and excellent mass transfer efficiency, along with spatial benefits. However, the authors also pointed out that the following issues require further investigation for better performance. Firstly, the formation and accumulation of LiOH from lithium metal may result in a lack of active lithium, which could affect the durability and efficiency of the system. Secondly, the use of ethanol as a proton donor negatively impacts the conductivity of the system. Thirdly, the system still requires humidification with THF, which does not entirely circumvent the necessity for a liquid electrolyte [26].

In light of the discussion above, it can be conjectured that the key to achieving further performance advantages in MEA designs may be found in the selection of proton donors that exhibit higher intermembrane mass transfer efficiency and greater compatibility. Besides, the selection of lithium salts is also a critical factor. Modulation of the SEI layer by anions has been demonstrated to enhance the stability of the lithium metal state and improve the durability of the system.

## 4 Design of Gel Electrolytes

Similar to polymer electrolytes, gel electrolytes also belong to non‐liquid electrolytes, yet probably possess higher ionic conductivity. Although the application of gel electrolytes in LiNR has not been reported so far, they have been extensively studied in lithium‐ion batteries, indicating that we can draw on these insights from battery science for LiNR electrolyte design.

The gel electrolytes consist of polymer substrates, liquid plasticizers, and lithium salts [27]. The polymer substrate acts as mechanical support, while the liquid plasticizer serves as an ion transport path, filling the voids in the polymer. The configuration renders the gel electrolyte near‐solid mechanical strength and near‐liquid ion transport capability. The superior mechanical strength hinders the growth of lithium dendrites to a certain extent and ensures the electrochemical response of the reaction through the maintenance of active lithium. Furthermore, the thickness of the gel polymer can be easily tailored to the micron level, contributing to compact configuration. Due to the excellent ductility of gels, they are compatible with electrode interfaces which gradually change during electrolysis. By selecting polymer materials with strong interaction with lithium [28], it is likely to mitigate the solid‐solid interfacial resistance observed by Cai et al. [26], therefore being conducive to higher energy efficiency.

The properties of gel electrolytes also make them suitable for multilayer structural designs. In the context of LiNR, these advantages can be harnessed by engineering multiple functional layers to decouple different design considerations. For instance, the layer in contact with the cathode can be tailored to promote the formation of a favorable SEI, while the transport layer can be optimized for ionic conductivity. Nonetheless, a potential drawback of gel electrolytes is their limited stability under high anodic potentials.

## 5 Construction of Dual‐Electrolytes System

The prevailing studies in LiNR are primarily concerned with the cathode aspect of the electrolytic system since the nitrogen reduction occurs at the cathode. Nevertheless, several optimizations from the anode perspective have been demonstrated to be necessary for continuous production of ammonia [14, 29]. For example, the design of viable HOR catalysts for the anode, which prevent the sacrifice of proton donor or solvent and significantly enhance the sustainability of the system. However, using the same electrolyte at both cathode and anode sides hampers customized optimization for the anode reaction. We assert that further decoupling of the cathode and anode reaction systems will broaden the spectrum of anode systems (such as, water splitting) that can be utilized, thereby making the reaction more controllable and simultaneously improving the economy of the chemical process.

Recently, Miao et al. developed an advanced dual‐electrolyte system for LiNR by integrating water electrolysis with a hydrophobic PTFE‐Nafion membrane and a polyoxometalate (POM, Li_3_PW_12_O_40_) proton shuttle. The membrane separates the organic catholyte from the aqueous anolyte, alleviating water crossover and lithium corrosion and thus achieving a faradaic efficiency of 54.2% [30]. Kim et al. reported a cross‐linked anion exchange membrane that is proved to be effective in suppressing water crossover from anolyte to catholyte during proton conduction, enabling stable NH_3_ generation with faradaic efficiency of about 60% [31]. In short, current research on dual‐electrolyte configurations is centered on rational design of separators. Although the separators slow the crossover of catholyte and anolyte, complete suppression is not achievable, posing challenges to their long‐term durability.

## 6 Conclusion

**FIGURE 2:**
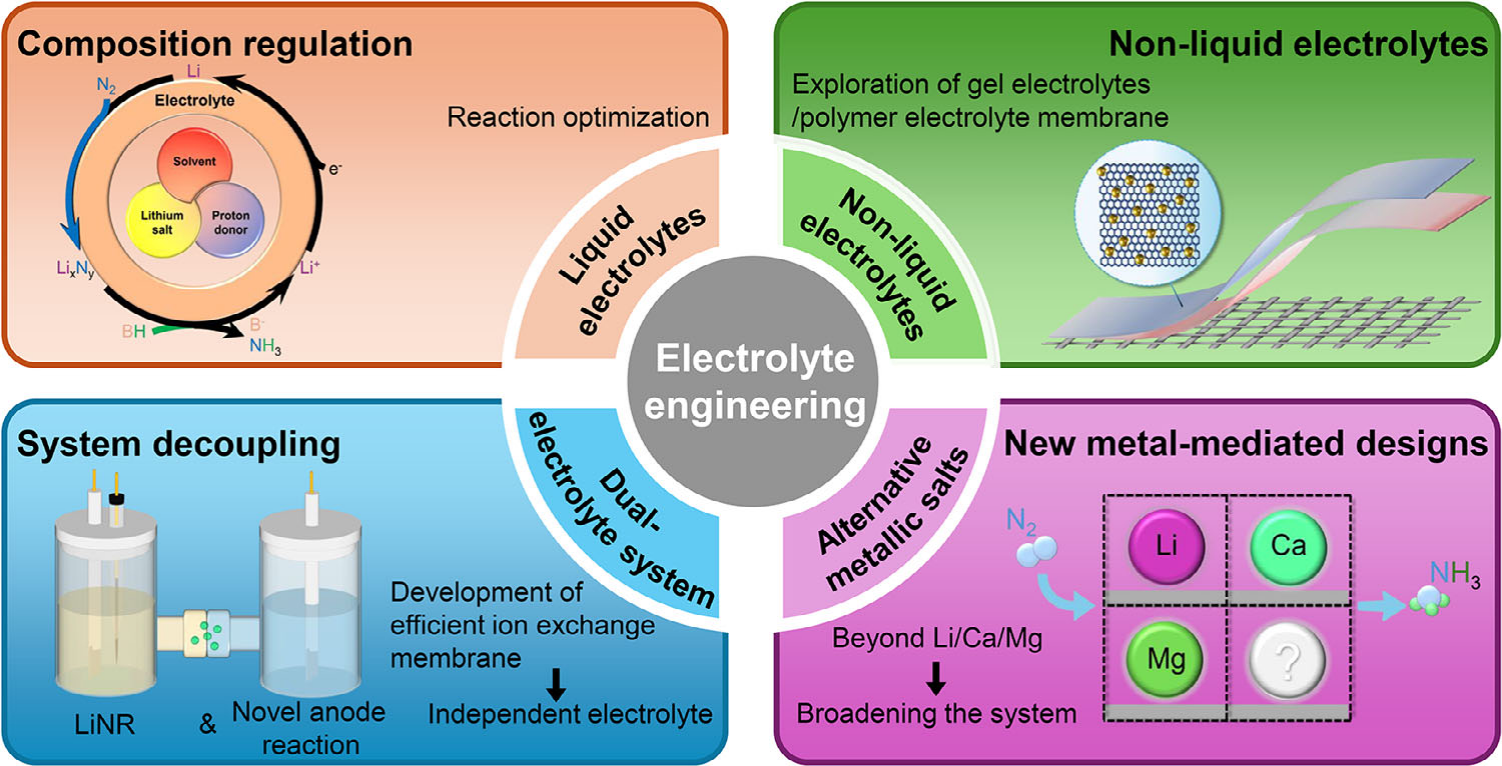
Electrolyte engineering strategies for future optimization of metal‐mediated nitrogen reduction systems.

In this perspective, we highlight the optimization of the LiNR systems through electrolyte engineering, as illustrated in Figure 2. Several strategies for different electrolyte systems have been proposed, including composition regulation, development of non‐liquid electrolytes, and decoupling the catholyte and anolyte (Table 1). Liquid electrolytes have been most intensively studied, yet still suffering from limited N_2_ transport and relatively bulky configurations. MEA and gel electrolytes hold promise in addressing these issues, but their practical applications are hampered by relatively low NH_3_ yield rates, limited faradaic efficiency, and poor stability.

**TABLE 1:** Characteristics of different types of electrolytes in LiNR.

Electrolyte	Liquid electrolytes [4, 14, 20, 21, 29, 32]	MEA [26]	Gel electrolytes^a^	Dual‐electrolytes [30, 31]
NH_3_ yield rate (µg h^−1^ cm^−2^)	20–15,000	30–50	10–100	10–600
Faradaic efficiency (%)	5–100	5–12	5–50	1–60
Cell voltage (V)	3.4–22	3.25 @ 5 mA cm^−2^	3.5–5	7–10
Pressure (atm)	1–15	∼1	1	∼1–20
Electrolyte thickness (mm)	∼3–20	0.12	0.1–1	∼3–30
Durability (h)	0.1–300	>3	1–50	3–50
Key Challenges	Limited N_2_ transportation, relatively bulky	Relatively low NH_3_ yield rate and faradaic efficiency	Limited stability	Electrolyte crossover and stability

^a^ As gel electrolytes have not yet been reported in LiNR research, the values listed in the table are presented as estimations.

Looking ahead, the development of electrolytes for LiNR should not only focus on improving the ammonia synthesis rate and faradaic efficiency, but also take into account practical considerations such as stability, durability, energy efficiency, economic viability, and compatibility with anodic reactions. For liquid electrolytes, MEA, and gel electrolytes, the key lies in developing ultrathin electrolytes that simultaneously show compatibility for both cathodic and anodic processes. The dual‐electrolyte systems offer greater flexibility for electrolytes design but encounter another challenge to explore separator with high ionic conductivity while effectively suppressing electrolyte crossover. We envisage that these insights and prospects can be applied to other metal‐mediated systems, broadening the future landscape of electrochemical ammonia production and enhancing its role in the future energy society.

## Author Contributions

**Xiyang Cai**: conceptualization, funding acquisition, writing – original draft, writing – review & editing. **Kaixiang Wang**: writing – original draft, writing – review & editing. **Ruilin Wang**: writing – review & editing. **Liuxuan Luo**: writing – original draft. **Shuiyun Shen**: project administration. **Guanghua Wei**: project administration. **Zhichuan J. Xu**: funding acquisition, writing – review & editing. **Junliang Zhang**: conceptualization, project administration, funding acquisition. All authors have read and approved the final version of the manuscript.

## Conflicts of Interest

The authors declare no conflicts of interest.

## Data Availability

Data will be made available on request.
